# α-Synuclein in Lewy Body Diseases: Progress, Remaining Challenges and Future Perspectives

**DOI:** 10.1017/erm.2026.10037

**Published:** 2026-02-18

**Authors:** Imogen J.H. Grimwade, Grey Enticknap, Eduardo De Pablo Fernandez, Cara Louise Croft

**Affiliations:** 1 Centre for Neuroscience, Surgery & Trauma, The Blizard Institute, Faculty of Medicine and Dentistry, Queen Mary University of London, UK; 2 University of Exeter, Exeter, UK; 3 Independent Researcher, UK; 4 Centre for Preventive Neurology, Faculty of Medicine and Dentistry, Queen Mary University of London, UK; 5 Queen Square Brain Bank for Neurological Disorders, UCL Queen Square Institute of Neurology, London, UK

**Keywords:** α-synuclein, dementia, Lewy body, Lewy body disease, neurodegeneration, Parkinson’s disease

## Abstract

Lewy bodies (LBs) are the main pathological feature of the neurodegenerative diseases Parkinson’s disease and Dementia with LBs. Since their discovery over 100 years ago, it is only in the last three decades, that, a wealth of genetic, pathological and pre-clinical evidence puts the spotlight on accumulated α-Synuclein (α-Syn) as the main component of LBs and implicated as a driver of these diseases. This has catapulted clinical trials for these diseases focussing on strategies to remove, reduce, disaggregate and prevent propagation of α-Syn. Advances in technical approaches have started to build a bigger picture of the complexity of LBs extending beyond α-Syn. There is still much to be learned about the processes underlying the formation and structure of LBs and their relationship to neurodegeneration. This will likely impact upon how we target these diseases therapeutically, diagnose them and build clinical trials. Here, we will discuss LBs in the context of α-Syn and other features, modelling strategies and how to direct research moving forwards in order to get clinical results. A more complete understanding of LBs and potential novel targets that drive their formation will likely lead to better outcomes in LB diseases.

## Introduction

Lewy body disease (LBD) encompasses the neurodegenerative disorders including Parkinson’s disease (PD), Parkinson’s disease dementia (PDD) and dementia with Lewy Bodies (DLB). Over 100 years ago, the pathological hallmark now known as a Lewy body (LB) was described by Friedrich Lewy, who discovered eosin-positive intracellular inclusions in post-mortem brain samples of his patients who had previously presented with motor-like symptoms and dementia (Ref. 1). The term LB was coined after Lewy himself by Lafora and Tretiakoff who reported an abundance of LBs in the substantia nigra (SN) of their PD patients, a few years later (Ref. 2).

With advances in microscopy, the ultrastructural features of LBs started to be recognised. A dense core surrounded by ordered filaments was first reported by Duffy and Tennyson in LBs of the SN and locus coeruleus using electron microscopy (Ref. 3). This is also accompanied by LB-type inclusions in neuronal processes in these areas, known as Lewy neurites (LNs) (Ref. 3). With the development of antibody approaches, LBs were also then identified to be neurofilament-, ubiquitin- and p62-positive (Refs 4, 5, 6). This was like other proteopathic inclusions identified in other neurodegenerative diseases such as Alzheimer’s disease (AD) albeit structurally different. In the late 90s, alongside genetic evidence of *SNCA* being linked to PD (Ref. 7), LBs were recognised by antibodies to α-Synuclein (α-Syn) (Refs 8, 9), and this led to LBDs being pursued as α-Synucleinopathies. Since then, a wealth of genetic, pathological and pre-clinical evidence has put α-Syn at the centre of these diseases and our knowledge of LBs. However, many open questions remain regarding the relationship of α-Syn, LBs, symptomatic presentation and associated neurodegeneration. Furthermore, as several clinical trials are already targeting α-Syn in LBDs it is important to understand how this targeting may affect disease course for patients.

Beyond this initial history, this review will evaluate our current knowledge of LBs and the role of α-Syn, how we model them and how to move forward from our biological findings on their formation and structure to clinical effectiveness in patients.

## Lewy body diseases (LBDs)

α-Synucleinopathies refers to a heterogeneous group of neurodegenerative disorders characterised by the presence of α-Syn inclusions and can be divided in those associated to LBs (comprising PD, PDD and DLB) and then Multiple System Atrophy (MSA), a distinct neurodegenerative disease with α-Syn glial cytoplasmic inclusions (GCIs) as the pathological hallmark.

PD, PDD and DLB are also classed as LBD and show substantial clinical and pathological overlap but also heterogeneity (Ref. 10). At post-mortem, PD, PDD and DLB cannot be distinguished on an individual level, with widespread LBs across the brainstem, limbic system and neocortex, with associated neurodegeneration in vulnerable regions (Ref. 11). Additionally, the use of cryo-electron microscopy demonstrated identical α-Syn filament structures in PD, PDD and LBD (Ref. 12).

Clinically, PD/PDD and DLB are characterised by a variable combination of parkinsonism and cognitive impairment classically divided by the timing of dementia onset in what is known as the 1-year rule; dementia may occur any time >1 year after the onset of motor symptoms in PD but occurs before or contemporaneously with motor symptoms in DLB. Due to the considerable clinical and pathological overlap, there is an ongoing debate whether PD/PDD and DLB represent distinct entities or different manifestations of a continuous spectrum (Refs 13, 14). Although some argue that this arbitrary cut-off may be useful in clinical practice to help plan care and prognosis, the presence of dementia was removed as an exclusion item in the clinical diagnostic criteria for PD (Ref. 15). The ongoing development of new biomarkers for the detection of different α-Syn conformations or alternative biomarkers may provide further light in the biological differences among PD/PDD and DLB (Ref. 16). This is particularly important for patients who want to appropriately plan their futures. Furthermore, work understanding the events that precede LB formation and extending reach beyond α-Syn detection may also facilitate accurate diagnosis and the future trajectory for patients.

## α-Synuclein

Genetic and pathological evidence linking α-Syn to PD emerged at a similar time, shifting research focus towards this LB constituent. Initial genetic evidence emerged in 1997, highlighting *SNCA* as a driver of familial PD (Ref. 7). Since this initial report, there have been substantial other reports genetically linking *SNCA* to PD, LBD and PDD (Refs 17, 18). In the same year, Spillantini et al. (Ref. 8) uncovered α-Syn as a major filamentous component of LBs with antibody staining in post-mortem PD brain and subsequently in DLB and PDD (Ref. 19) furthering the link between α-Syn and LBDs.

α-Syn is comprised of 140 amino acid residues (Ref. 20), has a molecular weight of around 14 kDa and has three distinct domains that pertain to its physiological functions as well as its potential pathology (Ref. 21). The N-terminal domain binds phospholipid membranes, including vesicles and presynaptic membranes (Refs 22–24), which are central to its role in neurotransmission (Ref. 25). The central domain enables fibrilization of α-Syn (Refs 26, 27), and the C-terminal domain has been identified as a key site for post-translational modifications (PTMs) (Ref. 28). In particular, the phosphorylation and truncation of α-Syn in this domain links closely to pathology (Ref. 29), with α-Syn in LBs being highly phosphorylated at Ser129 (Refs 9, 30). It should be noted that there is an emerging physiological role of pSer129 α-Syn (Ref. 25), particularly with regard to its reversible induction in response to neuronal activity (Ref. 31).

From the discovery of α-Syn in LBs, the study of the diseases PD, PDD, DLB and MSA and their grouping as α-Synucleinopathies (Ref. 32) has focused on a gain of toxic function by aggregated α-Syn (Ref. 33). This aggregated Lewy pathology and LBs are posited to progress in a stereotyped manner through vulnerable brain areas with several staging systems proposed based on this ‘prion-like’ progression (Refs 34, 35).

Extensive genetic evidence implicates *SNCA* with a causal role in familial PD, accounting for 10%–15% of cases (Refs 36, 37). *SNCA* multiplications have a dose-dependent effect on PD pathology, with triplications or homozygous duplications resulting in earlier onset and more severe disease phenotype, as well as higher penetrance, compared with heterozygous duplication or single gene carriers (Refs 38–41). Penetrance with triplications is almost complete (Ref. 42), although penetrance estimates with duplications range from 30% to 50% (Ref. 41). This increased penetrance may be due to increased expression of α-Syn caused by *SNCA* triplication (Ref. 38), although it is unclear how much expression differs between duplication and triplication carriers. This has added to the focus to the increased α-Syn driving a gain of function. This increased penetrance may also be due to other factors such as co-pathologies or other PD genetic risk, although this has not been explored yet. Various point mutations in *SNCA* have also been strongly linked to PD, including amino acid substitutions A18T, A29S, A30P, E46K, H50Q, G51D, A53T, A53V, A53E (Refs 36, 43) further highlighting the relationship of α-Syn to PD and LBDs. These mutations are associated with earlier onset and high penetrance, with the penetrance of A53T, for instance, being estimated between 85% and 90% (Refs 7, 44). Nonetheless, some A53T carriers remain disease free, and data surrounding the penetrance of other α-Syn variants remain less clear (Refs 7, 44, 45). Altogether, this suggests other potential contributors defining resilience or resistance to PD and LBs. Additionally, GWAS link common variants at the *SNCA* locus to sporadic PD, thus further supporting an important role of α-Syn in PD (Ref. 46).

Physiologically, numerous functions have been ascribed to α-Syn (47). On this basis, there has been some weight given to alternative thinking that its loss of function drives neurodegeneration in PD and that this should be a focus of therapies (Refs 33, 48). Neuronal and synaptic functions of α-Syn include neurotransmitter release and synaptic vesicle recycling and trafficking regulation (Refs 49–51). Further, in PD where the loss of dopaminergic neurons in the SN is a hallmark of the disease, the loss of α-Syn has been shown to drive dopaminergic neuron loss (Ref. 52) and drive neuroinflammation (Ref. 53). α-Syn is also required to sustain dopamine levels (Ref. 54). This is likely a complex relationship, as the loss of dopamine itself does not always drive dopaminergic neuron loss (Refs 54, 55). Further, α-Syn belongs in a family with other synuclein family members including β-synuclein and γ-synuclein which also show expression physiologically but also in PD and DLB (Refs 56–58). Removal of these alternative synuclein family members can also contribute further to dopamine loss and synaptic dysfunction (Refs 54, 59), suggesting differential compensatory effects across this family warranting more research into all synuclein family members.

## Insights from post-mortem LB studies

LBDs show significant overlap in clinical symptoms with other neurodegenerative disorders, particularly MSA and progressive supranuclear palsy (PSP) which are the diseases more commonly misdiagnosed in clinico-pathological series (Ref. 60). Currently, diagnosis is made clinically, and diagnostic confirmation can only be established with pathological assessment. Post-mortem LBs from various brain regions have received extensive study, and it is from this end-stage of disease that pre-clinical models have typically been developed to emulate (Ref. 61).

**Figure 1.**
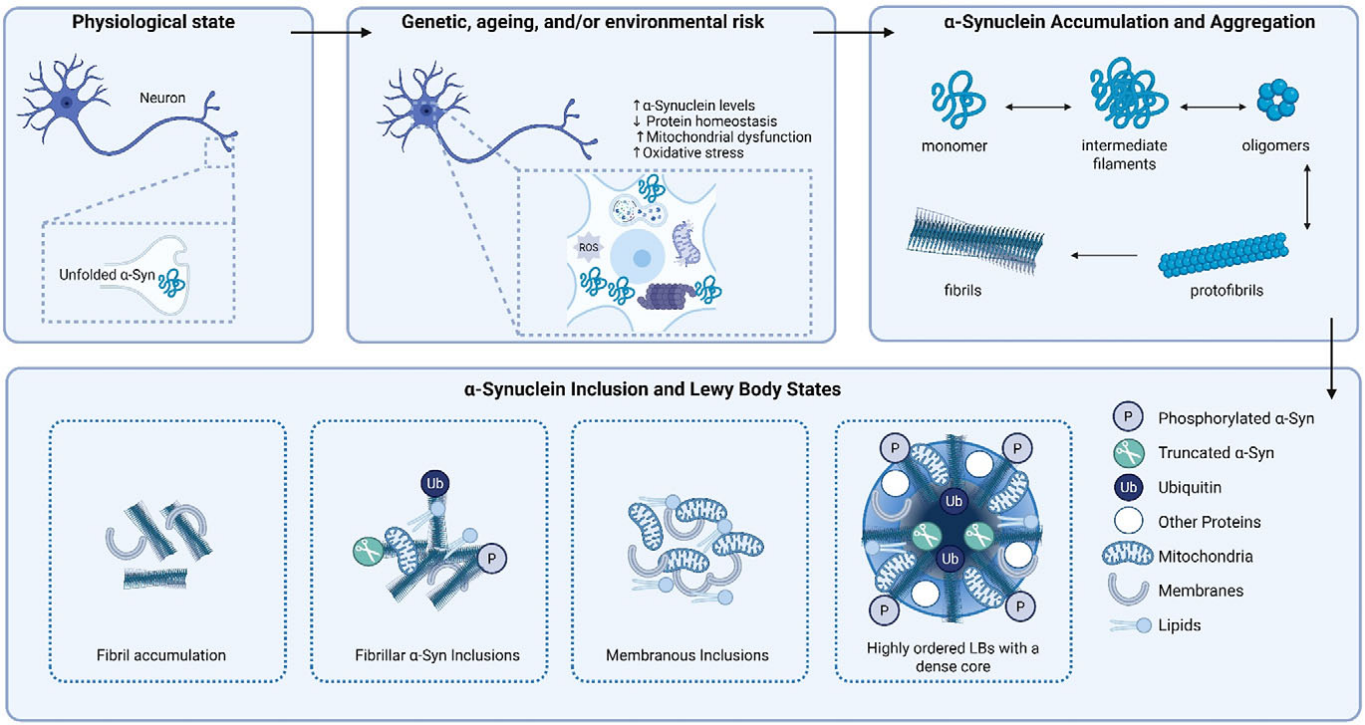
Pathways to α-Synuclein inclusion and Lewy body formation. In the physiological state, α-Synuclein (α-Syn) is typically found at the pre-synapse in its natively unfolded, monomeric state. Genetics, ageing and the environment can confer risk that leads to changes in α-Syn levels or structure, oxidative damage, mitochondrial dysfunction or impact lysosome/proteasome function to impair protein homeostasis pathways. This can drive the relocation of α-Syn to the somatic compartment where templated aggregation can be triggered typically on membranes. Here, α-Syn monomers undergo conformational changes to drive the formation of intermediate filaments that can assemble into oligomers. Oligomers then grow into protofibrils and assemble further into mature fibrils. These mature fibrils can then accumulate on membranes including those of lysosomes. As well as conformational changes, post-translational modifications of α-Syn including truncation, phosphorylation and ubiquitination can drive further aggregation on organellar membranes including mitochondria into fibrillar α-Syn inclusions also incorporating lipids with limited organisation. Membranous inclusions with limited fibrillar α-Syn are also formed containing clusters of mitochondria, organellar membranes and lipids. Over time, increased fibril growth on organellar membranes and post-translational modifications of α-Syn alongside other proteins transforms into a highly ordered, layered LB with a dense fibrillar core. Figure created with BioRender.com.

Post-mortem imaging-based studies of nigral LBs from PD patients have established a highly structured arrangement of different α-Syn species, with an electron-dense central core and distinct outer layers (Refs 62, 63). C-terminally truncated α-Syn alongside other lipids and proteins are predominant components of the core (Refs 62, 63). Peripheral to this, a layer of primarily pSer129 α-Syn is associated with other cytoskeletal elements (Refs 62, 63). Some regularity has been suggested around the C-terminally truncated and pSer129 α-Syn centrally of LBs. This delicate organisation may suggest that the formation of mature LBs is part of an organised and coordinated cellular response, particularly given that this type of LB is most often seen in later stage disease (Refs 62, 63). However, other LBs with less structure have also been identified (Refs 62, 63). More recent studies using correlative light electron microscopy (CLEM) also suggest a spectrum of LB structures some with, and some without, α-Syn fibrils (Refs 64, 65). Importantly, these studies recognise the heterogeneity of the LB and LBD brain pathology overall. Specifically, inclusions in the LBD brain can be fibrillar, membranous or a mixture of both. Furthermore, α-Syn can be found on membranous components in the soma or not at all (Refs 64, 65). Key events in α-Syn inclusion and LB formation are shown in Figure 1. Further insight into the importance of α-Syn has also been revealed through examining MSA brain. Although not commonly linked with the presence of robust LBs, studies of MSA brain by CLEM reveal the presence of α-Syn in neurons that is distinct from that found in oligodendrocytes in the disease but not classically appearing as LBs examined in other LBDs (Ref. 66). These differences may provide the basis of some heterogeneity. Furthermore, nuclear α-Syn is found in control brain tissue but is highly upregulated in DLB brain and is also phosphorylated at pSer129 (Ref. 67). This nuclear α-Syn is often found independently of LBs and may be homeostatically involved in nuclear transport, DNA damage response and DNA interactions (Refs 67, 68), suggesting further subcellular impacts and roles of α-Syn relevant to LBDs.

Beyond α-Syn, post-mortem studies have revealed that LBs contain various other lipids, organelles and proteins (Refs 61, 69, 70). As well as the α-Syn core in classical LBs, the enrichment of lipids and proteins in the core has also been highlighted (Refs 62, 69). In particular, the localisation of these lipid membranes, surrounding crowded mitochondria, vesicles and lysosomal-like structures (Ref. 57), highlights the potential role of these organelles in pathology beyond α-Syn. In particular, the wealth of mitochondrial and lysosomal risk genes already uncovered by PD GWAS (Ref. 46), as well as markers of mitochondrial, lysosomal and proteasomal dysfunction identified in PD brains, validate the involvement of these organelles in disease pathogenesis (Refs 64, 71, 72).

Proteomic studies of LBs from different brain regions and LBDs reveal the presence of over 200 other proteins, as well as over 40 tied to the presence of α-Syn (Refs 61, 73, 74). Proteins highly involved in signalling, apoptosis, the ubiquitin-proteasome system and several kinases are particularly enriched (Refs 73, 74). Future studies should determine whether these proteins of interest are bystanders or critical to the disease process in LBDs working in collaboration with, or isolated from, α-Syn.

The role of these proteins, lipids and disrupted membranes and membranous organelles in α-Syn aggregation and LB formation remains unclear but likely links to the heterogeneity of symptom presentation, onset and disease duration. Teasing apart these candidate molecules and proteins in future studies would inform the various stages of LB formation, maturation and the relationship to α-Syn and neurodegeneration.

## LBs, α-Synuclein and neurodegeneration

A big outstanding question is how α-Syn accumulation or LBs results in neurodegeneration. Addressing this will likely impact how we diagnose, prevent or treat LBDs. A substantial loss of dopaminergic innervation of the SN is required for clinical presentation of motor dysfunction in PD, and this loss is linked to onset and severity of symptoms although most likely with a non-linear association (Refs 75, 76). Additionally, there is evidence linking LB presence to neuronal death in the SN, with an estimated rate of production equal to the elimination of neurons containing them (Ref. 77). The presence of α-Syn in other brain regions also correlates well with levels of motor symptoms and visual hallucinations in LBD (Ref. 78).

On the other hand, correlation between LBs or α-Syn load with several clinical measures of disease severity such as disease duration, motor impairment and cognitive decline is less straightforward and highly variable across brain regions (Ref. 79). In the SN, where loss of dopaminergic neurons and nigral projections correlate with motor symptoms, the number of LB-bearing neurons remains stable throughout the disease, which has been interpreted as both proof of LB toxicity and lack of pathogenic role (Refs 77, 80). Adding to this disconnect, there is also evidence that neurodegeneration is not correlated with LB burden in the SN and that it may even precede LB formation (Refs 80, 81). On the genetic side, *SNCA* penetrance in duplication cases can be as low as 30% (Refs 41, 82). This low penetrance, clinical heterogeneity and absence of dopamine defects or neuronal loss in asymptomatic carriers suggest the complexity of α-Syn and its interaction with other genetic modifiers or environmental triggers in disease pathogenesis.

Interestingly, there is a known age-dependent increase in the prevalence of LBs later in life. This age-dependent increase surpasses the prevalence of PD or other LBDs by about three- to six-fold (Refs 83, 84), suggesting a disconnect between LBs, neuronal loss and symptomology.

Importantly, like some cognitively healthy individuals that can accumulate amyloid-β plaques without developing AD, LBs are found post-mortem in 10%–30% people without cognitive or motor impairment in what is known as incidental LBD (iLBD) (Refs 85–88). iLBD is highly suggestive of a pathological precursor of LBD rather than non-specific, age-related α-Syn deposition. This hypothesis is supported by different lines of evidence demonstrating a similar risk factor profile (Refs 89, 90) and an intermediate impairment between healthy individuals and people with LBD in dopaminergic nigral neurons, nigrostriatal projections, epicardial nerve fibre loss (Refs 91, 92, 93, 94). It is unclear if these individuals lived longer, whether they eventually would develop symptomatic disease or whether additional factors beyond Lewy pathology (genetic or otherwise) are implicated in the phenotypical presentation that can deem them resistant or resilient to the presence of LBs (Refs 85, 86).

Further evidence challenges the notion that LBs are a requisite for the diagnosis of PD. The diagnosis of PD in life remains clinical based on the presence of parkinsonism (a combination of bradykinesia plus resting tremor, rigidity or typical gait disturbances) in the appropriate clinical context (Ref. 15). Parkinsonism is a clinical syndrome not specific to LBDs and can be present in other neurodegenerative disorders in the absence of LBs and α-Syn at autopsy, although usually with features of neurodegeneration involving the SN (Refs 95, 96). On the other hand, LBs and SN degeneration can be found in rare neurodegenerative genetic disorders that present with a complex combination of neurological symptoms and signs not resembling PD (e.g., Niemann-Pick type C disease, *PLA2G6*-associated disorders (Refs 97, 98)). Some of the monogenic forms associated with PD (e.g., *LRRK2*, *Parkin*) present variable neuropathology which often do not include LBs (Refs 42, 99, 100). However, two recent independent studies have demonstrated widespread pathological oligomeric α-Syn aggregation without forming LB inclusions in cases with LRRK2-associated PD (Refs 101, 102). This was possible due to using a newly developed proximity ligation assay with increased sensitivity for oligomer detection compared to traditional LB immunohistochemical methods (Refs 101, 102). This may suggest that absence of LBs in some monogenic forms of PD may reflect the limitations of traditional detection methods, rather than a true absence of α-Syn. As such, this implicates early oligomeric α-Syn pathology with characteristic degeneration of the SN and clinical symptoms albeit without the need for LBs.

This disconnect between LBs and neurodegeneration may be a sign that an LB can sequester features which may otherwise prove harmful to a neuron. Supporting this, it appears that synaptic accumulation of α-Syn is particularly detrimental (Ref. 103). Pre-clinical experiments enable the staging of events in LB formation and will be discussed subsequently. Some pre-clinical evidence highlights that neurons are less vulnerable to α-Syn fibrillization or accumulation and that the recruitment of membraneless organelles, lipids and a more mature LB are linked to neuronal loss following synaptic and mitochondrial dysfunction or interaction (Refs 104, 105). This warrants further investigation particularly into organelle changes in LBDs that may be critical to disease.

The frequent disconnect between α-Syn, LBs and neurodegeneration suggests that LBs likely act as an indicator of the disease but simply highlighting their presence may fail to capture the complete and complex neurodegenerative processes which will need to be targeted therapeutically.

## Modelling α-synucleinopathy and LBs

Pre-clinical modelling facilitates some understanding of events preceding what is observed in end stage post-mortem brain. Here, we will summarise some of the cellular and animal models being used to investigate LBs, disease progression and neurodegeneration in LBDs (Refs 106, 107, 108).

Vulnerable cell types have been derived from induced pluripotent stem cells (iPSCs) developed from individuals with *SNCA* mutations or multiplications (Refs 109–113). 2D *SNCA*-A53T neuronal cultures develop axonal neuropathy, mitochondrial stress and substrate accumulation, as well as pSer129 α-Syn positive aggregates (Refs 110, 111). *SNCA*-triplication iPSC-derived dopaminergic neurons also show pSer129 α-Syn and some molecular features of PD at the transcriptomic level (Refs 112, 113). Using CLEM approaches, α-Syn has been shown to aggregate heterogeneously within *SNCA-*A53T iPSC-derived human neurons but preferentially on membrane surfaces (Ref. 105). This highlights α-Syn and seeding at membranes as likely important events in early LB formation.

To address the need for more cellularly diverse and spatially complex models, 3D midbrain and cortical organoids have also been established from *SNCA* triplication patient iPSCs. These show increased α-Syn levels and progressive α-Syn aggregation (Refs 114, 115) and can also incorporate non-neuronal cells (Ref. 116). These human systems begin to enable the study of α-Syn in disease but remain to show all characteristics of LBs.

*In vivo* models allow a wider consideration of the disease beyond the brain. A suite of transgenic and virally delivered α-Syn mouse and rat models have been developed exhibiting α-Syn-induced phenotypes (Ref. 117). The majority of transgenic mice overexpressing human wild-type or mutated α-Syn show intraneuronal α-Syn inclusions, some loss of striatal dopaminergic terminals, and some develop more mature LB features over time, such as electron-dense intranuclear deposits and ubiquitin-positive inclusions. However, these do not consistently show a fibrillar ultrastructure or neuronal loss in the SN (Refs 118–120).

Targeted recombinant adeno-associated virus (rAAV) vector delivery of human α-Syn (mutated or wild-type) to rodent and primate brains has also been used to study LBs (Ref. 121). This allows cell-specific modulation through different promoters, direct delivery to certain brain regions and different ages of injection (Refs 121–125). These models exhibit heterogeneous degenerative changes, including α-Syn aggregates, dystrophic neurites and dopaminergic neuronal loss (Ref. 122). Many show nigral α-Syn pathology (proteinase-K resistant and pSer129 α-Syn positive) and neurodegeneration and can also show PD-like α-Syn pathology in other relevant brain areas (Refs 121, 123–125). Therefore, rAAV α-Syn models provide flexible experimental opportunities to monitor progressive events linked to α-Syn overexpression and some wider LBD-like features but are yet to demonstrate mature LBs and have contrasting effects on neuronal loss (Refs 121, 123–125). Different rAAV injection paradigms and relationships to neurodegeneration are reviewed more extensively in Ref. (121).

Despite the focal importance of the SN in PD, LB pathology is found throughout the brain, proposed to spread gradually and sequentially according to several staging systems (Refs 126–128). Therefore, primary neurons, murine organotypic brain slice cultures and mice treated with α-Syn pre-formed fibrils (PFFs) have been widely used to study this seeding aspect (Refs 129–132). PFF-induced models can harbour features of LBs including pSer129 α-Syn, Thioflavin S-positivity, ubiquitination and fibrillar ultrastructures (Ref. 129). Neuritic pathology similar to LNs is also observed (Ref. 133). However, these LB-like aggregates generally lack the organisation and composition of mature LBs incorporating other proteins, lipids and organelles. This suggests PFFs typically model earlier stages of LB formation (Ref. 134) in the absence of extensive ultrastructural and biochemical analysis. However, more recently, PFF-treated rodent primary neurons have undergone extensive characterisation identifying structures with more commonality to human LBs including highly fibrillar structures, several PTMs and interactions with membranous organelles after longer term culture (Ref. 104). They also identified that fibrillar α-Syn accumulation is often marked by the classical LB markers including pSer129 α-Syn, ubiquitin and p62, suggesting that these markers align more with α-Syn rather than mature LBs. Notably, PFF-treated primary neurons exclude the presence of glia which may also play a key role in the disease. Extending from this, PFFs have also been applied to human iPSC-derived neurons in microfluidic devices facilitating assessment of α-Syn transport, as well as incorporating other CNS cell types to probe roles of glia (Refs 135, 136). PFFs have also been applied to *ex vivo* human brain slice cultures and in combination with rAAV A53T α-Syn overexpression can induce pSer129 positivity (Ref. 137). In sum, LB-like pathology of PFF models is often disconnected from neuronal loss and other LBD features, suggesting that fibrillar α-Syn alone may not be capable of the full pathology induction of LBDs (Ref. 134). However, these model systems enable valuable exploration of early α-Syn induced changes otherwise not feasible in living patients.

Rather than focussing on modelling LBs, others have prioritised studying neuronal loss in the SN. Models based on the injection of neurotoxins such as MPTP in rodents and non-human primates have yielded the nigrostriatal loss of idiopathic PD (Refs 138–140). These models typically show degeneration without α-Syn pathological features; however, some longer-term treatments show some α-Syn accumulation (Refs 138–140). Notably, there has been large variation in nigral loss, striatal dopamine loss and associated behavioural deficits following both short- and long-term treatment in these models. These models are unable to address the disparity between α-Syn inclusions and degeneration but may be complementary to a more complete understanding of LBDs.

Genetic studies have implicated other genes beyond *SNCA* for PD and other LBDs. *LRRK2* is one such gene where *LRRK2* deficiency abolishes toxic features induced by nigral α-Syn overexpression (Ref. 141), although others have shown limited ability of LRRK2 to modulate α-Syn (Ref. 142). In other *LRRK2* models, varying degrees of SN degeneration have been identified with some developing mild α-Syn accumulation (Refs 143, 144). As clinical PD patients with a *LRRK2* mutation also show early α-Syn pathology rather than LB pathology post-mortem with consistent SN neuronal loss (Refs 42, 101, 102, 145, 146), then these models may be valuable to understand PD beyond LBs. Conversely to *LRRK2* carriers, nearly all PD patients with a *GBA1* mutation have LBs. *GBA1* mutations have also been associated with other α-Synucleinopathies like LBD (Ref. 42). However, the extent of neuronal loss in *GBA1*-focussed models is unclear as most have been developed expressing mutations in *SNCA* too (Ref. 147).

To date, the pre-clinical models have clearly mimicked α-Syn pathology and nigral loss, but challenges arise from the limited ultrastructural characterisation presented in many models as to whether mature LBs incorporating other proteins, lipids and organelles are present. Future models will likely push towards being able to comprehensively bring together characteristic LBs, degeneration and the motor and cognitive symptoms in a single model. Some caution should also be advised as many models describe mild to modest neurodegeneration driving behavioural deficits, whereas in humans, a substantial loss of the SN is required before symptoms present. By addressing this disparity beyond potential species-specific differences would complement our understanding of this disease aspect.

## Arguments against focussing on α-Syn as the key player in the pathological process

The disconnect between LBs, α-Syn and neurodegeneration evaluated earlier provides one of the biggest arguments that targeting α-Syn in LBs may not impact disease progression and symptomatic presentation.

The propagation and spread of α-Syn has received considerable study due to several staging systems based on post-mortem LB presence suggesting ‘prion-like’ progression (Refs 34, 35). This has directed a great deal of translational focus to stopping the spread of α-Syn (Ref. 33) despite most α-Syn being found intracellularly. As there is still no dynamic data on the progression of LBs or α-Syn pathology from individuals with PD to support or contend this narrative, this should be addressed in future studies. Novel approaches and specific α-Syn PET tracers to evaluate this longitudinally in the same individuals would be welcomed.

Importantly, beyond *SNCA* as a PD-related gene, there are several other genes linked to PD, many of which are involved in autophagy-lysosomal pathways, or ubiquitin-proteosome pathways, pointing towards disordered proteostasis as a key player in PD (Ref. 46). These include *GBA1* and *LRRK2* which both play a role in autophagy-lysosomal pathways, with mutations in either linked to perturbed protein clearance and subsequent accumulation of α-Syn (Refs 148, 149). *PRKN* encodes a ubiquitin E3 ligase, promoting mitophagy and proteasomal degradation of α-Syn, and subsequently mutations in this gene may promote accumulation of α-Syn and damaged mitochondria (Ref. 150). The roles of these genes suggest that the pathogenesis of PD may in part be a dysfunction of proteostasis occurring upstream of any α-Syn or LB pathology. Additionally, other mitochondrial genes confer PD risk, strongly implicating mitochondrial dysfunction as a key driver of pathogenesis. Mitochondrial involvement in PD appears to span diverse mechanisms, including dysfunction of mitochondrial quality control and clearance, as well as defects in mitochondrial protein production (Ref. 151). PD is therefore not solely a disorder of α-Syn as there are many genes implicating wider organelle involvement, which may be driving disease and symptoms. When summarising the discordance of PD genetics and LBs, there are three key themes: genes that link to PD but do not always have LB pathology, genes that link to PD and LBs, but also other pathologies are present, and non-PD syndromes that show LB-like pathology, e.g., Krabbe’s disease (Ref. 152). Unravelling this further will likely lead to a greater understanding of LBs themselves, as well as PD or other LBDs.

Furthermore, some pre-clinical data suggest α-Syn expression as potentially having a neuroprotective role in response to injury. Following the induction of apoptosis in neurons in rats, α-Syn is upregulated potentially as a compensatory response in surviving neurons (Ref. 153). Neuronal overexpression of α-Syn is also protective against paraquat neurotoxicity in mice (Ref. 154). Intriguingly, α-Syn expression is high in surviving dopaminergic neurons in the SN in PD, although this change could be a selective survival or upregulation due to PD progression as is seen in duplication or triplication familial PD cases (Ref. 155). However, these cases of α-Syn as neuroprotective are limited in the field. Paradoxically, LB distribution or density is not associated with the severity of nigral cell loss with the presence of more LBs associating with more nigral neurons (Ref. 80) and that neuronal loss may precede LB formation (Refs 80, 81). Similarly, α-Syn aggregation and the formation of inclusions have also been demonstrated to be a protective process in pre-clinical models (Refs 156, 157), suggesting that the field requires further understanding of which, if any, α-Syn species are more critical than others. Likewise, the loss of physiological α-Syn has received some discourse, and it could well be that the loss of soluble α-Syn is key to disease (Ref. 33). Adding to this, a small pathological study highlights a decrease in levels of soluble α-Syn in the progression of PD alongside greater abundance of LBs (Ref. 158).

As previously discussed, LBs are comprised of various proteins, lipids and organelles (Refs 64, 65, 74). It could well be that any or several of these act alone or in concert with α-Syn to drive degeneration and symptoms. Further research in this area should evaluate this hypothesis. In particular, the role of lipids has received limited study, but their interaction with α-Syn and other LB constituents may likely reveal novel therapeutic targets (Ref. 159). Additionally, the emergence of mixed pathologies and potential synergistic relationships between different disease features will also likely inform our future understanding.

Similarly, there has been a great focus on pSer129 α-Syn in pathological studies of LBD brain, yet recent work brings emerging roles of pSer129 α-Syn phosphorylation critical to its physiological function (Ref. 160). Similarly, pSer129 α-Syn increases during neuronal activity and environmental enrichment (Ref. 31), and it is not a prerequisite for α-Syn spread or aggregation (Refs 132, 161). Historically, this marker has been used by pathologists worldwide to denote a LBD upon post-mortem brain analyses (Refs 19, 30), but we are only just learning the flexible nature of this marker. Shifting focus from pSer129 α-Syn to other α-Syn species or targets could enable greater disease understanding by looking at other potential markers for future optimisation.

## Translational implications and clinical applications

To date, there are currently 9 trials of disease-modifying therapies with α-Syn as a target registered out of a total 60 disease-modifying therapy registered trials in PD (Ref. 162), with 76 other trials registered for symptomatic therapies. At least for PD, the disease-modifying therapeutic pipeline also takes into account approaches targeting *GBA1* and *LRRK2* and targets for inflammation, neuroprotection and mitochondrial function (Ref. 162). As we aim for therapies that slow, prevent or cure LBDs, it is likely that looking beyond α-Syn as a target either towards other LB constituents or alternative biological pathways implicated in LBDs may be therapeutically worthwhile at least in some case of LBDs.

We have already discussed how there is evidence for and against the relationship of α-Syn in LBs with neurodegeneration and LBD symptoms. As trial data emerge, it will be interesting to see whether therapies with α-Syn as a target are beneficial. Importantly, as we still have limited knowledge of the physiological role of α-Syn, strategies that lower α-Syn may also require caution, particularly in the context of some pre-clinical data suggesting the loss of α-Syn can be damaging (Refs 52, 53) and that monomeric α-Syn is already reduced in PD (Ref. 158).

The finding that the templated amplification of α-Syn monomer with seed competent α-Syn from patient biofluid samples allows a strong differentiation between the presence of PD and healthy controls implicates the potential use of Seed Amplification Assays (SAAs) in clinical practice and trial stratification (Ref. 163). This is caveated with the complexity that the SAA does not detect *LRRK2* or *GBA1* carriers with PD accurately, suggesting disconnect between α-Syn and LBDs (Ref. 163). Similarly, many diagnosed with PSP or Corticobasal Syndrome show SAA positivity (Ref. 164), which may highlight the presence and importance of co-pathologies but could also lead to misdiagnosis or mis-inclusion in clinical trials. Further reliance on SAA positivity as a marker of LBDs could also lead to certain non-positive patients being excluded from an accurate diagnosis and clinical trial inclusion particularly as approximately 1 in 10 patients clinically diagnosed with PD have negative α-Syn SAAs (Ref. 165).

Since its initial report, the identification of PD has relied on a clinician only diagnosis which can then only be verified upon post-mortem analysis (Ref. 60). As with any disease, there is often a surge to push towards the biological detection of the disease. This has recently led to two somewhat differing biological frameworks for the future classification and diagnosis of PD. The first heavily focuses on α-Syn (Ref. 166), with a positive SAA strongly encouraged in the neuronal α-Syn disease integrated staging system (NSD-ISS). Notably, this excludes any genetic PD beyond *SNCA* cases. The second staging system recommended for PD is the SynNeurGe three-component system taking into account the presence of α-Syn (Syn), evidence of neurodegeneration (Neur) and genetic predisposition (Ge) (Ref. 167). Both incorporate α-Syn as a critical part of these frameworks meaning that α-Syn will be further cemented in the diagnosis of PD if a consensus is reached. Furthermore, as the α-Syn SAA is susceptible to some false positives, some healthy individuals would ‘biologically’ have PD but never show symptoms or develop disease.

On the translational side, as we have alluded to earlier, it is important that clinical trial outcomes are not affected or skewed by prioritising α-Syn SAA. This could strongly impact eligibility for clinical trial participation. Furthermore, it is not clear whether α-Syn SAA positivity can be reversed by therapies, particularly as it is a binary measure, so it is unclear how this would then be used in disease-modifying trials or whether traditional clinical metrics be reverted to at this point.

It is clear in the AD field that PET tracers and other biomarkers for Amyloid-β and tau have facilitated the improvement of clinical trials leading to the development of the first approved Amyloid-β immunotherapies (Ref. 168). Future work should ensure more longitudinal analysis of LBD biological disease features whether that be α-Syn PET or alternative tracers, biomarkers or metrics. Although no α-Syn PET tracers are yet in clinics, promising recent results indicate some progress in this space, but further improvements to sensitivity will likely be required to document PD progression (Ref. 169).

Lastly, across the neurodegeneration field, it is clear that many cases contain mixed pathologies or develop alongside other comorbidities (Ref. 78). It is likely that these mixed pathologies contribute to the heterogeneity of symptoms in patients and should receive further study to improve possibilities to treat these cases (Refs 78, 170). Notably, in those diagnosed with PD, the mean ‘co-pathologies’ found in a large cohort study stands at 3 per individual, suggesting opportunity to understand how these contribute to disease progression or modulation (Ref. 171).

## Furthering our understanding of α-Syn and LBs in disease

Looking forward, there are several areas to focus on, which should elevate our understanding of where to direct effort for therapeutic development and confirm if α-Syn and LBs are suitable targets in LBDs.

On the α-Syn side, it is clear that there are gaps in our knowledge around, which, if any, species of α-Syn are toxic and should be targeted. Also, we need to understand the processes involved in the production of different α-Syn species and the eventual formation of α-Syn-containing LBs. Importantly, as novel methodologies and technologies advance, then this may also reframe our previous knowledge and thinking. For example, new approaches have enabled detection of smaller α-Syn species in brains that were otherwise deemed α-Syn free with traditional methods (Refs 101, 102) and super-resolution imaging approaches now facilitate detection of nanoscale aggregates of α-Syn in post-mortem brain (Ref. 172). These will be critical to better understanding these processes. This goes alongside understanding how, and if, these link to neuronal dysfunction and degeneration. We should also be increasing our understanding of the physiological role of α-Syn and, in particular, further examination of pSer129 α-Syn in physiology or the loss of function hypothesis of α-Syn (Refs 25, 33). Similarly, like most diseases of the brain, initial study has adopted a neuron-centric view of the disease; however, LBDs are multisystem disorders involving multiple brain regions and non-neuronal cell types, as well as other organs and a potential role for the immune system (Ref. 173). Understanding dysfunction beyond the neuron may also provide novel insights and drug targets.

Other than α-Syn, it will be important to determine the relationship with other synucleins and how knockout and compensation works amongst family members. In particular, a SNP in *SNCG* has recently been found in two cases of motor neuron disease and is also amyloidogenic *in vitro* (Ref. 174) implicating other synucleins in neurodegenerative disease.

When it comes to LBs, future studies to determine the biochemical composition and how different components of LBs may modulate disease are critical. It is likely that this and understanding more about their histological and ultrastructural morphologies and better modelling of them may provide novel angles to target to LBDs. This may also play in to the heterogeneous presentation of symptoms and disease course. Likewise, the field has limited knowledge around LNs and their contribution to disease. It could be this form of pathology that is more disease critical.

We have briefly covered the state of play in pre-clinical modelling of α-Syn pathology and potential LBD features; however, it is yet to be determined if we truly recapitulate LBs and the preceding stages in our pre-clinical models. Evaluating any novel models alongside more robust biochemical, morphological and structural examination should position us for future success. Importantly, pre-clinical models will be our main hope to spatiotemporally evaluate the sequence of events that drive the formation and evolution of LBs and LNs and for future therapeutic evaluation.

In critiquing pre-clinical models, we regularly focus on how they differ from end-stage disease post-mortem brain. At this point, these brains are essentially in brain organ failure, so it is not surprising that there is limited overlap. The field has speculated the initiation of pathological processes and the subsequent impact on brain function and symptomology from late-stage static data so we should be open to the fact that features of our pre-clinical models may represent the earlier truth. In contrast, the full progression of disease cannot be studied post-mortem due to little opportunity to gain longitudinal information from an individual so approaches that can evaluate longitudinal development of disease or the formation and maturation of LBs in humans will also be much welcomed.

On the genetic side, as substantial risk identified by GWAS converges on autophagy-lysosomal function, proteasomal or mitochondrial function (Ref. 46), this warrants further understanding of these biological pathways and their potential contribution to PD and potentially other LBDs. Unravelling at what stage lysosomal, proteasomal or mitochondrial dysfunction occurs in pathogenesis will be crucial in understanding how these organelles are involved as cause or consequence of disease.

Cases of iLBD are suggestive of early LBD with limited degeneration and no symptomatic presentation. As more of these cases emerge, it may be suitable to probe them to ascertain whether if they had survived longer, they would also succumb to disease and determine some of the earliest phases of disease in humans. Alternatively, they may possess factors that confer resilience or resistance to the effects of α-Syn and LBs which could potentially form the basis of treatments. Likewise, as many of those with REM sleep behaviour disorder go on to develop α-Synucleinopathies (Ref. 175), this also presents an interesting group of individuals to expand study and potentially identify earlier disease features.

## Summary

Most LBD patients when receiving a diagnosis want to know their personal disease trajectory. As it stands, the nature of LBDs is heterogeneous with no clear or predictable course. Coupled with that, most cases received limited, if any, acute symptomatic relief and no curative therapies are available yet. This leaves LBD patients with an unclear picture of what their precise future disease and life looks like.

As trial outcomes are reported, in particular the 9 trials with α-Syn as a target and the 51 other disease-modifying therapy trials in PD (Ref. 162), we hope a more complete picture of the suitability of α-Syn for targeting will emerge; however, this will always be contingent on the correct trial design with the inclusion of the right patients, with the right therapies at the right time.

At present in the field, α-Syn remains a clear focus particularly due to the pre-clinical, genetic and pathological evidence accumulated to date. However, we should remain open-minded to what the presence of α-Syn in LBs means and whether other pathways may be more therapeutically fruitful. We also should focus efforts on understanding the events that precede LB formation. This may enable more accurate diagnosis through identification of early molecular or cellular markers and identify novel therapeutic targets to provide a more certain personal disease trajectory for patients.

## Data Availability

No datasets were generated or analysed during the study.

## Abbreviations

α-Syn: α-synuclein
AD: Alzheimer’s disease
CLEM: correlative light electron microscopy
DLB: dementia with Lewy Bodies
GCI: glial cytoplasmic inclusions
iLBD: incidental Lewy Body Disease
iPSC: induced pluripotent stem cell
LB: Lewy body
LBD: Lewy body disease
LN: Lewy neurite
MSA: multiple system atrophy
NSD-ISS: neuronal α-Syn disease integrated staging system
PD: Parkinson’s disease
PDD: Parkinson’s disease with dementia
PFF: pre-formed fibrils
PSP: progressive supranuclear palsy
PTM: post-translational modification
rAAV: recombinant adeno-associated virus
SAA: seed amplification assay
SN: substantia nigra
